# Authors’ Response to Peer Reviews of “Development of a Digital Platform to Promote Mother and Child Health in Underserved Areas of a Lower-Middle-Income Country: Mixed Methods Formative Study”

**DOI:** 10.2196/60266

**Published:** 2024-07-31

**Authors:** Zaeem Ul Haq, Ayesha Naeem, Durayya Zaeem, Mohina Sohail, Noor ul Ain Pervaiz

**Affiliations:** 1 Gavi, The Vaccine Alliance, Islamabad, Pakistan; 2 Health Services Academy, Islamabad, Pakistan; 3 Onto Global, Islamabad, Pakistan; 4 Alliance for Behavioural Communication & Development, Islamabad, Pakistan

**Keywords:** primary health care, mother and child health, community health worker, slums, digital applications, health communication.

*This is the authors’ response to peer-review reports for “Development of a Digital Platform to Promote Mother and Child Health in Underserved Areas of a Lower-Middle-Income Country: Mixed Methods Formative Study.”*

## Round 1 Review

### Reviewer BM [1]

#### General Comments

*This study [* *2* *] draws on multiple sources of data to assess the efficacy and feasibility of a video-based mobile health (mHealth) tablet intervention used to train and equip 10 community health workers (CHWs) in two slums in Pakistan. The overall strength of the paper is that the authors have collected in-depth qualitative data that can help inform the field on how to build and distribute such an intervention to improve the needs in low-resource communities. The paper should be strengthened by pinpointing the unique and new contributions of the study findings to inform the field on digital health education interventions for CHWs. While the intervention is described in detail, more work is needed to explicate why this study expands our understanding of digital health education for CHWs in deprived settings.*

Response: Thank you very much for appreciating the strengths of our study and helping us identify the weaknesses. In the revised version, we have reframed our paper in the context of communication capacity building of the CHWs serving within the primary health care system in low- and middle-income countries. We explicate with the help of data from the digital form of IEC materials, which are more interesting to the audience, improves fidelity during the implementation, and is not very costly.

#### Specific Comments

##### Major Comments

*1. What the authors have found is largely expected and demonstrated in other work globally. Not surprisingly, they find that there is a severe lack of knowledge and critical need for education among CHWs and their patients to bring about behaviors that can improve maternal child health in low-resource communities. It is also not surprising that a well-resourced, highly supported, small-scale pilot (of just 10 CHWs trained) would be successful. While these findings are all important to describe in detail (as the authors have done), to provide more value in this field of work, I suggest the paper more explicitly emphasize what contribution the study adds to the literature. What are the new and important takeaways to improve how mHealth education interventions for CHWs are developed?*

Response: We appreciate this comment as it helped us position our findings in a better way. We realize that a small sample of health workers would show better results when they receive focused training (Discussion section, page 12, lines 18‐20). In the revised version we highlight that our CHWs did not have prior training or experience of working in the health sector. So, their absorption of the knowledge and fidelity to the intervention is meaningful (Discussion section, page 11, line 48 onwards). We also highlight that using behavior change theory provides a structure to the home visits and keeps the health worker and their audience attentive (page 11, lines 20‐27), while the videos ensure consistency of the content across all health providers. That we were able to produce the videos and the digital app on a low budget is also an important takeaway that we highlight in the Methods (page 6, lines 26 and 27) and Discussion (page 11, lines 39‐42) sections.

*2. In addition to more explicitly pointing to the contributions of their findings, the paper could be strengthened considerably by a discussion of how their findings can inform how this small-scale pilot can be taken to scale effectively. The authors allude to this, but I think more could be added with regard to cost-effectiveness. It sounds like an expensive and involved intervention—to my understanding, providing a tablet to CHWs, hosting a two-day training, overseeing an apprentice week, and a refresher training, all on top of development of 14 videos and a calendar for patients. Information on the costs to develop and implement this intervention could be better described, and I would appreciate a critical lens on what would be needed to scale, including identification of barriers. I think the limitations they described with respect to CHW availability, connectivity, etc, should be folded into this discussion. I think this would set up well the ongoing work they describe to test real-world effectiveness in 250 mother-infant pairs.*

Response: Thank you for this helpful comment; it made us realize that we need to include more information on costs, and what it really means for Sehat Ghar. The development of the videos in the application was not very costly in our case, and we have included this information in the intervention description (page 6, lines 26 and 27) and discussed its implications in the Discussion section (page 11, line 39). In fact, our observation during this study and afterward is that with most smartphones, recording and editing videos are not a problem, and a number of young creative professionals are available who can do this on a low budget. Being primarily a health education intervention, our application did not need installations of updates or uploading of data from the field, which minimized its dependence on high-speed internet. We have discussed these advantages as well as the challenges to upscale in the Discussion section (page 12, lines 25‐32).

*3. A corollary to the above comment, because the intervention involves so many moving parts (ie, provision of a device, development of videos, in-person training and supervision), do their findings point to particular components of the intervention that are particularly important?*

Response: Thanks for this insight. Sehat Ghar is a “whole” that is comprised of many “parts,” and it is difficult to tease out a few to be the most valuable. Moreover, we were not looking for critical elements of our intervention during the formative phase and lack data to make a substantive statement. However, in the last two paragraphs of the Discussion section (page 12), we have indicated that something can be initiated through community members in areas that are totally ignored by the public sector. Likewise, videos might be one element that could be disseminated via several media.

*4. The introduction of the paper starts by highlighting the distinctions between the definitions of inequality, disparity, and inequity. I don’t think the comparison adds any value to the introduction. In fact, I was confused because, after the first paragraph, implications of an expensive mHealth intervention for equity are not discussed at all. Just because a study is conducted in a low-resource setting, does not mean that it is working to improve equity. If the authors want to focus on equity, I would appreciate a more critical lens on how the high costs of a digital intervention met with barriers like internet connectivity improve the situation of the poorest communities. (Otherwise, I suggest changing the introduction paragraph.) A video-based tablet intervention that relies upon internet could ostensibly be more effective in communities with more infrastructure and resources, and when scaled more widely in better resourced communities, digital interventions may actually broaden the gap between the haves and the have-nots. How can we think about ways digital interventions can be implemented to ensure this does not happen? (Is this why the in-person CHW-to-patient link is so important? Can this be unpacked?)*

Response: This is a very valuable insight, which made us rethink our current paper. We realize that we are working with a marginalized community, and our work will contribute to not only improving their behaviors in the household but also helping them get better engagement from the health system. However, this can make sense when we have the data from the rollout of the intervention to the 250 families. For now, therefore, we have removed the context of inequity and situated our work in the context of the lack of primary health care services and the communication capacity building of health workers (Introduction, page 3).

*5. The phase two findings draw heavily upon the “qualitative feedback” obtained from CHWs and mothers about the Sehat Ghar application and tablet use, but there are scant details on how these data were collected and analyzed. If these qualitative findings are so prominent in the results and not merely anecdotal and complementary to other findings, they need to be described with the level of detail the preintervention in-depth interviews and focus group discussions were described. Were semistructured guides used? What framework was applied to the development of qualitative protocols? How were the data coded and analyzed? How many CHWs and mothers participated?*

Response: Thank you for this comment. Indeed, the data collection and analysis needed more organization and clarity because of the several phases involved in this formative study. In the revised submission, we have included an overarching question (page 5, lines 14-16) that served as a framework. We have included more details about data collection tools and methods along with clarifying qualitative and quantitative analysis for all three phases of the study (page 5, sections on data collection and data management and analysis).

*6. How were the two slums selected for focus? What inclusion/exclusion criteria, if any, were applied when thinking about geographic selection? How do the two slums generalize to the larger set of slums in Islamabad?*

Response: Thanks for indicating this requirement, which we have fulfilled by including the selection criteria in the Methods section. At this stage, we were focused on improving health behaviors at the household level including the improvement in health seeking for mothers and children from the formal health system. So, we had to select clusters close to the public sector health facility. Including some financial support would be required for traveling for those slums that are far; however, providing transportation was not within the remit of this project. We aim to include financial support in the next iterations of our study.

*7. More of a description of the health systems in the slums is helpful for readers who are not familiar. How do CHWs fit into the larger health system? To my understanding, the 15 CHWs that were included in this study were completely new to the profession as “we identified volunteer women willing to become CHWs.” Why focus on completely new volunteers for the study rather than drawing upon the existing CHW workforce? Is it because there were not CHWs already at work in these areas? If there are other CHWs already serving these slums, can this be better described? Please also speak to the generalizability of the findings given that the CHWs in this study started with a much lower lack of knowledge given their novice status. If the study were to be done with experienced CHWs, perhaps the delta in knowledge gains would not be nearly as large.*

Response: These are very good points; thank you. The lady health workers of Pakistan’s national program primarily work in rural areas. Slums are ignored, and these two from Islamabad also belong to the same category. We describe this lack of primary health coverage in the first part of the Methods section (page 4, lines 2-8). We also agree that the large gradient of knowledge improvement that we achieved may have been due to the novice CHWs. We acknowledge this limitation and some of the challenges that this intervention may face with the lady health workers of the national program in the Discussion section (page 12, lines 16-33).

*8. A more detailed description of how participants (health care providers, CHWs, and mothers) were recruited for the study is needed. What was the sampling frame? What were the inclusion/exclusion criteria? What was the consent rate? What roles did the health care providers hold (ie, were they doctors, nurses, other roles)?*

Response: We have made information about all these categories more explicit in the Study Participants section (page 5, lines 2-13).

*9. What were the protocols for conducting observations? Were they unannounced or were CHWs prepared in advance to know that the supervisors would be conducting the observations? Can you address limitations with respect to bias, as individuals generally behave quite differently when observed?*

Response: Thanks for this question. We have included the relevant information in the Data Collection section (page 5, lines 23-29) and discussed the limitations (page 12, lines 21-29), as these are important.

*10. Do the authors have any analytics (ie, frequency of video views, engagement with the app) from the tablet/application that can be used to support the observation data and qualitative feedback on the intervention feasibility?*

Response: Thanks for this question. Using digital analytics was not within the project scope and hence it was not considered during the formative phase.

##### Minor Comments

*1. In the Abstract, identify the larger geographic location of the communities.*

Response: Thanks. We have included the geographical location in the first paragraph of the Abstract.

*2. Is there a more recent citation than the 2015 reference used for [3]?*

Response: We have replaced this with the most recent reference from Census 2023 of Pakistan [4].

*3. The Methods section says that the initial five transcripts were coded independently by two members of the team. What about the remaining transcripts? Were there any checks/reconciliation on the coding of the remaining transcripts?*

Response: The initial transcripts were used to develop a code list, which the two researchers discussed and finalized. The final code list was used for analyzing all transcripts (page 5, lines 37-41).

*4. Consider moving some of the details of the intervention, including on page 7 of the Results, to the Methods section. When reading the Methods, I expected to see more of these details there and am a bit confused as to why they are included in the Results.*

Response: Thanks for this observation. It helped us in reorganizing the Methods and Results sections. In the revised version, we have moved the intervention details to the Methods section with an independent subheading “The Intervention” (page 6).

*5. Suggest not paraphrasing Steve Jobs in the Discussion section.*

Response: Thanks, we have deleted that part in the revised submission.

*6. The manuscript states that this pilot was conducted in 2018. The study also notes that ongoing work with 250 mother-infant pairs is currently being conducted, now 5 years later. Given how much has happened in the world, I am curious if the authors have any reflections on how the pandemic has changed the way we should understand and reflect their findings. (The pandemic need not be addressed in the manuscript, but the second to last paragraph of the Discussion talks about health emergencies. I am skeptical how such an involved pilot could be so quickly mobilized to respond to health emergencies. The authors should reflect on this if they believe findings point to this as a possibility. I also think the detailed statistics about flooding in Pakistan and other emergencies are out of scope for the paper. I don’t believe anyone needs convincing that health emergencies of this nature exist.)*

Response: Thanks for this valuable input. We have deleted the content about emergencies and floods, and included our reflections on how this study might contribute to response efforts in outbreak situations (page 12, lines 11-19).

### Anonymous [5]

#### General Comments

*This paper addresses a principal issue, especially for the developing world where the valuable lives of mothers and children can easily be prevented. However, of course, a big challenge in the proposed solution is the availability of Android devices that are also connected to the internet. This is a limitation, therefore, to be added. Another area that needs to be addressed is related to cultural acceptance and sensitivity to using technology, particularly during the prenatal stage. Also, I noted that the diagrams are not clearly developed and placed in the appropriate places. I am happy with the qualitative part but unfortunately not an authority on quantitative; thus, this part should be vetted by a quantitative expert.*

Response: Thanks very much for appreciating the value this study brings to the lives of mothers and children living in underserved areas.

- In the revised version, we have mentioned the costs of development (which were not much) and the occasional need for internet, as data were not required to upload or download while in the field.
- In our discussions with participant mothers and the community, we did not come across any myths or apprehensions about using digital technology while being pregnant.
- We have revised our diagram and placed it in the appropriate place, as advised.

#### Specific Comments

##### Major Comments

*1. This is an excellent topic requiring continuous literature development.*

Response: Thank you for appreciating our contribution.

*2. The method used is mixed, whereas I would have preferred the total qualitative inquiry considering the set aims and objectives.*

Response: Thanks for this observation. We relied mostly on qualitative methods and have used quantitative only where it could improve the robustness of the study.

*3. The authors need to pay attention to the sociocultural realities of the context; therefore, either address them or acknowledge them as limitations.*

Response: Thanks for this important observation. Our biggest constraining reality was the absence of a formally engaged CHW from the public sector, which we circumvented by engaging volunteers. Second, we did not propose biomedical steps (eg, frequent ultrasound or going to health facility for each and every problem) that would entail high costs for the family. Rather, we empowered them with knowledge and skills to identify and troubleshoot problems that could be addressed at home and have a new understanding that something can be done even in the worst circumstances. These are small bits of value, expected to help women and their families living in an underserved context.

##### Minor Comments

*4. The diagrams need to be appropriately designed and placed in the paper.*

Response: Thanks for this input. We have reduced our diagrams to one and have placed it within the text (page 4) as advised.

## Round 2 Review

### Reviewer BM

*The authors have very thoughtfully and substantially revised the paper, making clear the methods and contributions of the study. I appreciate the detail with which the authors pointed to their edits in the revised manuscript and am satisfied with their changes.*

Response: Thank you very much for appreciating the effort we made in revising the draft. We were able to considerably improve it because of the extensive input you gave—thanks again!

*At this point, I suggest only very minor revisions, asking authors to check grammar and conduct a copyedit of the paper. There are instances where a careful copyedit will improve the overall reading experience of the paper. For instance, in the Abstract, I suggest the following changes:*

1. *Drop the “the” in “Can the information-technology (IT) help these CHWs?”*
2. *Add a comma after “application” in “We explored answers through development and feasibility testing of Sehat Ghar, an android-based digital application to improve the communication capacity of volunteer CHWs in two slums of Islamabad.”*
3. *Do not capitalize “Focus Group Discussions” in the Methods section.*

Response: In light of your overarching comment about grammar and copyediting, we have revised the entire manuscript including the three specific observations you made in the Abstract:

1. We have dropped “the” in line 6 of the Abstract.
2. Added a comma after “application” in line 8 of the Abstract.
3. Dropped capitalization of “Focus Group Discussions” in line 13 of the Abstract.

We hope that our effort in improving the language and grammar has resulted in a better expression, overall, and it meets the required standards. We are highly indebted to your kind time and valuable inputs and wish to close our response with a sincere thanks once again.

Kind regards.

## Abbreviations

CHW: community health worker
mHealth: mobile health
